# Obstructive sleep apnea, intermittent hypoxemia and prothrombotic biomarkers

**DOI:** 10.5935/1984-0063.20190147

**Published:** 2020

**Authors:** Kunal Deokar, Sushant Meshram, Gopal Chawla, Nana Kunjir, Chetna Meshram, Nupur Abrol, Priyanka Gaikwad

**Affiliations:** 1 Government medical college, Pulmonary Medicine - Nagpur - Maharashtra - India.; 2 All India Institute of Medical Sciences, Pulmonary Medicine and Sleep Disorders - New Delhi - Delhi - India.; 3 Government medical college, Pharmacology - Nagpur - Maharashtra - India.; 4 All India Institute of Medical Sciences, Anaesthesia - Delhi - Delhi - India.; 5 Bhaktivedanta hospital, Paediatrics - Mumbai - Maharashtra - India.

**Keywords:** Sleep Apnea Syndrome, Fibrinogen, Hypoxia

## Abstract

**Objective:**

To study the serum levels of ﬁbrinogen and d-dimer in patients with obstructive sleep apnea (OSA) and its correlation with apnea hypopnea index (AHI), oxygen desaturation index (ODI), minimal oxygen saturation and arousal index.

**Methods:**

It was a case control study in which 23 cases of OSA and 23 controls were enrolled. Morning fasting serum ﬁbrinogen and d-dimer were measured in cases of OSA and controls.

**Results:**

Serum ﬁbrinogen levels among OSA patients (268.47±53.11mg/dl) were elevated as compared to the levels in controls (221.52±65.84mg/dl) (p<0.05). Serum ﬁbrinogen co-related positively with AHI (r=0.6381, p=0.0011) and ODI (r=0.7434, p=0.0000), negatively with minimal oxygen saturation (r=-0.4461, p=0.0329). There was no statistically signiﬁcant correlation of ﬁbrinogen with arousal index (r=0.2697, p=0.2133). There was no statistically signiﬁcant difference between mean fasting d-dimer level in cases (0.12mg/L, 0.06±0.18mg/L) and controls (0.12mg/L, 0.02±0.22mg/L) (p=0.8926).

**Conclusions:**

The observation of elevated ﬁbrinogen levels with the increasing severity of OSA and hypoxemic events makes OSA one of the important risk factor for cardiovascular disorders.

## INTRODUCTION

The risk of coronary artery disease and cerebrovascular disease is more in patients with obstructive sleep apnea^1,2^. Obstructive sleep apnea is characterized by repetitive episodes of complete or partial upper airway collapse resulting in intermittent hypoxemia and inflammation. Intermittent hypoxemia probably leads to increased sympathetic activity ^3^. Hypoxia-reoxygenation leads to the generation of free radicals resulting in oxidative stress which along with the resultant endothelial dysfunction may trigger the coagulation cascade.

There have been various biomarkers which have been studied in the past linking cardiovascular diseases with OSA. Some biomarkers like Brain Natriuretic peptide (BNP) were associated with good results but are affected by age and weight. Thus, it is necessary to limit its role as an ideal biomarker in cases of OSA where the majority of patients are obese. Whereas other biomarkers like cardiac troponin, interleukin 6 and C reactive proteins are associated with variable results.^4-7^

Plasma fibrinogen plays a very important role in the coagulation cascade. It has been proven to be a major determinant of blood viscosity and blood flow. High plasma fibrinogen levels were associated with an increased risk of cardiovascular disease. Several epidemiological studies have shown that elevated plasma fibrinogen levels are independently related to the development of vascular disease^8,9^. D- Dimer indicates activation of coagulation and downstream fibrinolysis. Elevations in d-dimer are considered to be a procoagulant harbinger of a hypercoagulable state^10^.

We hypothesized that OSA might confer a hypercoagulable state due to intermittent nocturnal hypoxemia inducing oxidative stress, and thus could be an independent risk factor for cardiovascular and cerebrovascular episodes. Even though several studies have tried to assess the relationship between fibrinogen with OSA, the results have been inconsistent. Some studies have found that fibrinogen is elevated in OSA^11-14^ while in a study by von Känel R et al ^15^ it was not elevated. Also, the above studies did not adjust their results for various factors like COPD, smoking, stroke etc. in which fibrinogen could be elevated. With this background, this study was undertaken to determine the levels of fibrinogen and d-dimer in patients with obstructive sleep apnea.

## MATERIAL AND METHODS

Study design: Case-control study

Patient recruitment: The patients of obstructive sleep apnea were selected from the sleep clinic of the same institute. The study was carried out after approval from the institutional ethics committee and with fully informed written consent from the subjects. The patients were divided into the following groups:

Cases: Those with symptoms suggestive of OSA like snoring, daytime sleepiness, witnessed breathing interruptions during sleep, Epworth sleepiness score >10 and AHI ≥ 5 on polysomnography. (n = 23)

Controls: Healthy individuals with no symptoms suggestive of OSA like snoring, daytime sleepiness, witnessed breathing interruptions during sleep, Epworth sleepiness score<10 and AHI < 5 on polysomnography. (n = 23)

Those with liver disease, drug intake - stimulants, sedatives, oral anticoagulants, aspirin, oral contraceptives, estrogen replacement therapy; current alcoholics (someone, who at the time of the study, drinks alcohol either daily or occasionally); hypertension (blood pressure > 140 mm Hg or diastolic blood pressure > 90 mm Hg) or if using any antihypertensive medication ^16^; diabetes mellitus (fasting glucose > 126 mg/dl or oral glucose tolerance test > 200 mg/dl or use of hypoglycemic medication) ^17^; those with history of angina, myocardial infarction, coronary heart disease, stroke, heart failure and/or who have undergone coronary artery bypass graft surgery or carotid endarterectomy; smokers (someone, who at the time of study smokes any tobacco product either daily or occasionally as per the classification criteria suggested by WHO) ^18^ and diagnosed cases of COPD were excluded from the study.

For both cases and control groups, various anthropometric measurements like height, weight and neck circumference at the upper border of the cricothyroid membrane in an upright position were measured. Body mass index was calculated. Pulmonary function tests were done on the same day between 10.00 am to 02.00 pm. The patient was then given detailed instructions to follow for polysomnography at 08:00 pm. Early in the morning, fasting blood samples were taken and sent for investigations. Polysomnography was performed using Alice 5 (Philips Respironics, Murrysville, Pennsylvania, United States). PSG consisted of continuous polygraphic recording from surface leads for electroencephalography, electro-oculography, electromyography, electrocardiography, pressure transducer, and thermal flow sensor for nasal airflow, thoracic and abdominal impedance belts for respiratory effort, pulse oximetry, microphone for snoring, and sensors for body position. Our centre was 310m above sea level. PSG scoring for sleep stages and respiratory events like apnoea and hypopnea was performed following the recommendations outlined in the AASM manual ^19^. The average number of desaturation episodes per hour were measured using PSG and were called the oxygen desaturation index (ODI). Desaturation episodes were generally described as a decrease in the mean oxygen saturation of ≥4% (over the last 120 seconds) that lasted for at least 10 seconds. Venous blood was sampled at the same centre between 7:00 and 8:00 am in the supine position the next morning after overnight polysomnography and an overnight fast for various biochemical testing. Fibrinogen was measured by using the Clauss method^20^ and D-dimer was measured by using immunofiltration and sandwich-type method at the same centre where PSG was performed^21,22^.

### Statistical analysis:

Continuous variables were presented as mean ± SD; categorical variables were expressed in actual numbers and percentages. Data was checked for normality before statistical analysis by using the Shapiro-Wilk test. Serum fibrinogen was compared between cases and controls by performing unpaired t-test. Categorical variables were compared by chi-square statistics. Strength and magnitude of correlation of serum fibrinogen with other study variables was assessed by computing Pearson’s correlation coefficient (r). All the tests were 2 sided. p - value < 0.05 was considered as statistically significant. Statistical software STATA version 10.0 was used for statistical analysis

## RESULTS

In total 131 cases were screened for the present study. Of these 53 patients had either diabetes, hypertension or both thus were excluded. 39 were smoker with or without chronic obstructive pulmonary disease and 16 others who had a history of ischemic heart disease were also excluded. In total 23 patients who fulfilled inclusion criteria were enrolled in the study and analyzed. The mean age (in years) of cases and controls were 49.39 ± 12.30 and 48.08 ± 11.64 respectively. In both the groups, 17 (74%) were males and 16 (26%) were females. There was no significant difference in weight (in kg.) of individuals between the two groups (Cases vs. Controls: 81.62 ± 15.71 kg; vs. 81.82 ± 14.21 kg) (p=0.9641). There was no significant difference in BMI of individuals across the two groups (p=0.8346). There was no significant difference in FVC (Cases vs. Controls: 2.88 ± 0.78 L; vs. 2.98 ± 0.89 L) (p=0.8) and FEV1 (Cases vs. Controls: 2.28 ± 0.69 L; vs. 2.3 ± 0.72 L) (p=0.96) between the two groups (Table1).

**Table 1:** Age and BMI distribution among cases and controls.

Parameter	Cases	Controls	p
	Mean ± SD	Mean ± SD	
Age(yrs)	49.39 ± 12.30	48.08 ± 11.64	0.7137
BMI (kg/m2)	30.25 ± 4.73	29.95 ± 5.06	0.8346

**Figure 1:**
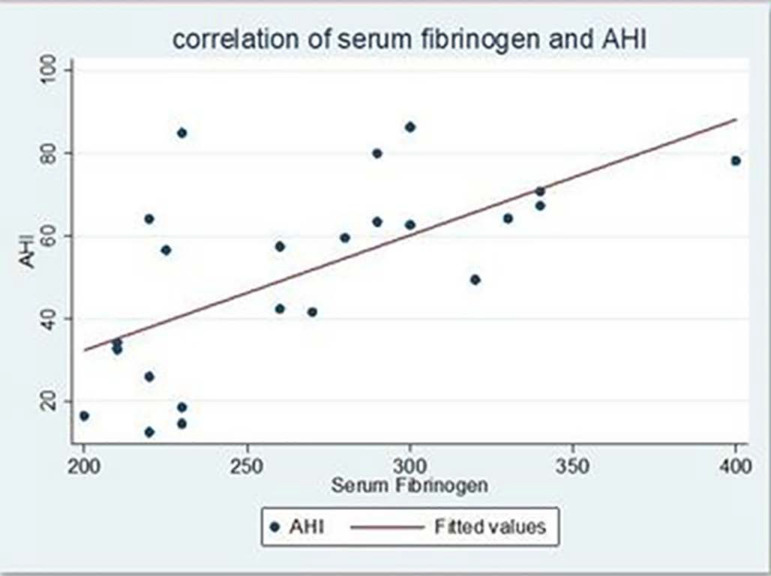
Correlation of fasting serum fibrinogen with AHI.

**Figure 2:**
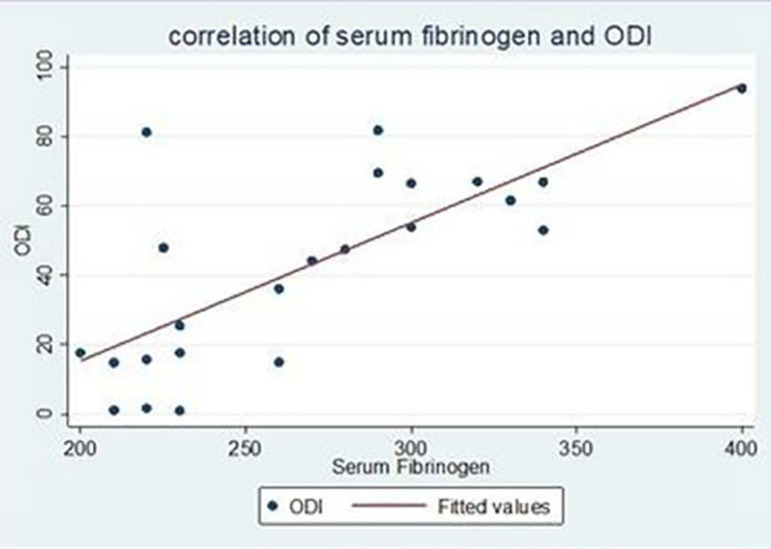
Correlation of fasting serum fibrinogen with ODI.

Most of our cases had AHI more than 30/hour (n=18). There were 3 cases with AHI between 5 to 15/hour and 2 cases with AHI between 16 to 30/hour. Most of our cases had ODI more than 30/hour (n = 14). There were 3 cases with ODI less than 5/hour and 6 cases with ODI between 15 to 30/hour. The mean fasting serum fibrinogen level was more in cases (268.47 ± 53.11 mg/dl) as compared to controls (221.52 ± 65.84 mg/dl). This difference was statistically significant (p=0.0108). There was a moderate to strong correlation between fasting fibrinogen and apnea-hypopnea index (r = 0.6381, p = 0.0011) (Figure1) which was also statistically significant. Thus, with an increase in AHI, fasting serum fibrinogen levels also increased. Similarly, there was a statistically significant strong correlation between fasting serum fibrinogen levels and oxygen desaturation index (r = 0.7434, p = 0.0000) (Figure2). There was a negative correlation between minimal oxygen saturation and fasting serum fibrinogen (r = -04461, p = 0.0329) which was statistically significant. Similarly, there was a statistically significant negative correlation between fasting serum fibrinogen levels and average minimal oxygen saturation (r = -0.6176, p = 0.0017). There was a weak positive correlation between arousal index and fasting serum fibrinogen (r = 0.2697, p = 0.2133), however, it was not statistically significant. (Table 2)

**Table 2:** Correlation of fasting serum fibrinogen with various polysomnographic indices.

Variable	Fasting serum fibrinogen	
	r-value	p-value
Apnea-hypopnea index	0.6381	0.0011
Oxygen desaturation index	0.7434	0.0000
Minimal oxygen saturation	-0.4461	0.0329
Average minimal oxygen saturation	-0.6176	0.0017
Arousal index	0.2697	0.2133

There was no statistically significant difference between mean fasting d-dimer level in cases (0.12 mg/L, 0.06 ± 0.18 mg/L) and controls (0.12 mg/L, 0.02 ± 0.22 mg/L) (p = 0.8926). A positive correlation of d-dimer with AHI (r = 0.1509, p = 0.4920) and ODI (r = 0.3472, p = 0.1042) was observed, however they were not statistically significant. A weak negative correlation of d-dimer was observed with minimal oxygen saturation (r = -0.1775) and average minimal oxygen saturation (r =-0.1454, p = 0.5079) but they were also not statistically significant. There was no correlation of d-dimer with arousal index (r = 0.0592, p = 0.7884). (Table 3)

**Table 3:** Correlation of d-dimer with various polysomnographic indices.

Variable	Fasting d-dimer	
	r-value	p-value
Apnea-hypopnea index	0.1509	0.4920
Oxygen desaturation index	0.3472	0.1042
Minimal oxygen desaturation	-0.1775	0.4178
Average minimal oxygen saturation	-0.1454	0.5079
Arousal index	0.0592	0.7884

## DISCUSSION

The major finding of our study was that the average fasting serum fibrinogen levels among OSA patients (268.47 ± 53.11 mg/dl) were elevated as compared to the levels in controls (221.52 ± 65.84 mg/dl) which was statistically significant (p value < 0.05). Barceló A et al ^11^ has shown that the serum fibrinogen levels in children with OSA are elevated as compared to controls. Steiner S et al ^12^ has also shown that OSA is associated with higher plasma fibrinogen (353 ± 83 mg/dl vs. 317 ± 62 mg/dl, p = 0.015).

In our study, serum fibrinogen in patients with OSA co-related positively with apnea-hypopnea index (r = 0.6381, p = 0.0011) and oxygen desaturation index (r = 0.7434, p = 0.0000). A negative correlation of fasting serum fibrinogen with minimal oxygen saturation (r = -0.4461, p = 0.0329) and average minimal oxygen saturation (r = -0.6176, p = 0.0017) was also seen. There was no statistically significant co-relation of fasting serum fibrinogen levels with arousal index (r = 0.2697, p = 0.2133). In a study by Steiner S et al ^12^, serum fibrinogen correlated with nocturnal minimal oxygen saturation (r = - 0.275, p = 0.0036) and AHI (r = 0.297, p = 0.001). In patients of ischemic stroke with OSA, Wessendorf et al ^13^ showed that fibrinogen levels positively correlated with RDI (r = 0.24, p = 0.007) and negatively correlated with several oxygen indices including average minimal oxygen saturation (r = - 0.41, p < 0.001). Bouloukaki et al ^14^ in a cross-sectional study, showed that fibrinogen was elevated in OSA patients as compared to controls. Furthermore, several experimental studies have shown that the severity of intermittent nocturnal hypoxemia may contribute to procoagulant disturbances in OSA, which is consistent with our findings of correlation of elevated fibrinogen levels with hypoxemic indices^23-28^.

Though arousals are associated with bursts of sympathetic activity, not all arousals are associated with airway obstruction and intermittent hypoxemia ^29^. And probably, therefore, there was no correlation between arousal index and fibrinogen. The finding of an association between AHI and hypoxemic indices with fibrinogen suggests that severity of intermittent nocturnal hypoxemia may contribute to procoagulant disturbances in OSA. We noted that as the severity of obstructive sleep apnea increases, there is an increase in the fibrinogen levels, which in turn may increase the risk of adverse cardiovascular events. Thus, our findings are consistent with most clinic-based samples that have reported an increased risk of adverse cardiometabolic outcomes at the severe levels of OSA.

D-dimer levels in patients with OSA were not elevated as compared to the controls and the difference between them was not statistically significant (0.12 ± 0.06 mg/L vs. 0.12 ± 0.10 mg/L, p = 0.8926). There was a weak positive correlation of D-dimer with AHI (r = 0.1509, p = 0.4920), positive correlation with ODI (r = 0.3472, p = 0.1042), weak negative correlation with minimal oxygen desaturation (r = -0.1775, p = 0.4178) and average minimal oxygen desaturation, (r =-0.1454, p = 0.5079) but none of them were statistically significant. There was no correlation of d-dimer with arousal index (r = 0.0592, p = 0.7884). In a study by Mehra et al there was no significant relationship between d-dimer and AHI or arousal index ^30^. Von Kanel et al studied the association between polysomnographic measures of disrupted sleep and prothrombotic factors and found that there was no association of d-dimer with AHI ^31^. D-dimer is formed after the breakdown of a well-formed fibrin clot. Therefore, probably we did not observe any increase of d-dimer in patients with OSA.

Inferences regarding correlations of serum fibrinogen with various study variables were based on a relatively modest sample size. Most of the OSA patients attending our tertiary care centre suffered from co-morbid conditions like hypertension, diabetes mellitus, stroke, heart failure, myocardial infarction. As per our exclusion criteria, we could not include these patients with co-morbidities, which limited our sample size. We tried to exclude many known confounders; residual confounding factors nonetheless may have influenced our results. As we observed that ODI correlated better with fibrinogen, further studies may evaluate the relation between percentage of time spent at various oxygen saturation levels with fibrinogen.

## CONCLUSION

Raised serum fibrinogen levels in patients with obstructive sleep apnea are suggestive of a procoagulant state that might provide an explanatory link for the high prevalence of vascular diseases in these patients. Therefore, early diagnosis and treatment of obstructive sleep apnea is warranted to prevent its cardio-cerebrovascular consequences.

Since we observed a correlation of fibrinogen with AHI and hypoxemic indices, it is possible that the oxidative stress may be a reason for procoagulant state in OSA. Serum fibrinogen may be used as a biomarker to assess cardiovascular risk in OSA. The efficacy of the drugs, which reduce serum fibrinogen level, can be evaluated in the patients of obstructive sleep apnea to minimize cardio-cerebrovascular morbidity and mortality.
